# Cytomorphology of Circulating Colorectal Tumor Cells:A Small Case Series

**DOI:** 10.1155/2010/861341

**Published:** 2010-01-06

**Authors:** Dena Marrinucci, Kelly Bethel, Daniel Lazar, Jennifer Fisher, Edward Huynh, Peter Clark, Richard Bruce, Jorge Nieva, Peter Kuhn

**Affiliations:** ^1^Cell Biology Department, The Scripps Research Institute, 10550 North Torrey Pines Road, La Jolla, CA 92037, USA; ^2^Scripps Clinic Medical Group, Scripps Clinic, 10666 North Torrey Pines Road, La Jolla, CA 92037, USA; ^3^Scripps-PARC Institute for Advanced Biomedical Sciences, Palo Alto Research Center, 3333 Coyote Hill Road, Palo Alto, CA 94304, USA; ^4^Oncology & Hematology Department, Billings Clinic, 2825 Eighth Avenue North, Billings, MT 59107, USA

## Abstract

Several methodologies exist to enumerate circulating tumor cells (CTCs) from the blood of cancer patients; however, most methodologies lack high-resolution imaging, and thus, little is known about the cytomorphologic features of these cells. In this study of metastatic colorectal cancer patients, we used immunofluorescent staining with fiber-optic array scanning technology to identify CTCs, with subsequent Wright-Giemsa and Papanicolau staining. The CTCs were compared to the corresponding primary and metastatic tumors. The colorectal CTCs showed marked intrapatient pleomorphism. In comparison to the corresponding tissue biopsies, cells from all sites showed similar pleomorphism, demonstrating that colorectal CTCs retain the pleomorphism present in regions of solid growth. They also often retain particular cytomorphologic features present in the patient's primary and/or metastatic tumor tissue. This study provides an initial analysis of the cytomorphologic features of circulating colon cancer cells, providing a foundation for further investigation into the significance and metastatic potential of CTCs.

## 1. Introduction

The spread of cancer cells from the primary tumor site to distant organs results in an incurable condition associated with a high incidence of mortality [1]. Evidence indicates that primary tumor cells gain access to the bloodstream, thus becoming circulating tumor cells (CTCs) that travel via the peripheral blood to sites anatomically distant from the primary tumor. There they form secondary tumors, eventually producing lethal metastases, the major cause of treatment failure in cancer patients [2, 3]. 

Circulating epithelial cells (CEpiCs) have been detected in the peripheral blood of patients with a variety of metastatic epithelial malignancies at varying concentrations using several different methodologies [4–10]. In general, CEpiCs can be identified (using immunofluorescence assays) via monoclonal antibodies directed against epithelial-specific antigens, allowing them to be distinguished from normal blood cells [11, 12]. In the setting of a patient with known metastatic carcinoma, these circulating epithelial cells are generally presumed to be circulating malignant tumor cells by most researchers in the field. Support for this presumption is emerging; research has demonstrated genomic changes of single CEpiCs and that the cells are aneuploid, suggestive evidence that these cells are indeed malignant [13–16]. For the purpose of this study, we use cytologic features of the CEpiCs and their correlation with the primary biopsies to support their presumed malignancy and henceforth in this report refer to these cells as CTCs.

The presence of CTCs has been shown to correlate with poor prognosis and lower survival in metastatic breast and colorectal cancer patients [17–20]. Limited pilot studies have also indicated the utility of detecting CTCs in metastatic colorectal patients although there is apparent discrepancy in enumeration among various studies [4, 20, 21]. CTCs provide the link between the primary and metastatic tumors [22] so identification and characterization of CTCs holds promising implications for the detection and treatment management of metastatic epithelial malignancies. Furthermore, isolation and characterization of these cells will provide new insights on the biological mechanisms of metastasis. 

Most CTC detection methods depend on immunomagnetic separation and immunofluorescent labeling of epithelial-specific antigens such as epithelial cell adhesion molecule (EpCam) and cytokeratin (CK); however, because of methodologic limitations, cytologic details of the cells are not discernible, and detailed morphologic studies and images of CTCs are limited [13, 22, 23]. Detailed description and images of the morphologic types of CTCs found specifically in metastatic colorectal patients are even more scant [23]. 

Much remains unknown about circulating tumor cells: how they enter the bloodstream, how frequently they are destroyed within the bloodstream, how they exit the bloodstream, and whether each CTC has the same metastatic potential, that is, the same potential for extravasation and development into a new tumor in a metastatic site [24]. Thus, mere enumeration of CTCs must be augmented by the ability to study individual cells by additional morphologic and/or molecular characterization that could provide clinically useful information as well as aid in our understanding of the metastatic process.

We have previously reported about an enrichment-free immunofluorescent staining protocol with fiber-optic array scanning technology (FAST) to enumerate and characterize CTCs found in metastatic cancer patients [5, 22, 25]. The methodology allows for detailed cytomorphologic analysis, and we have previously reported a detailed review of circulating breast carcinoma cells. We now add circulating colon cancer to the limited atlas of CTCs, with this small series in which we cytomorphologically evaluate CTCs from the blood of five metastatic colorectal cancer patients. Single cells found in the blood are also compared to archived histopathologic and cytologic specimens of the patient's primary and/or metastatic tumor.

## 2. Materials and Methods

### 2.1. Collection of Blood Samples

Five metastatic colorectal cancer patients provided informed consent at Scripps Clinic (La Jolla, CA) as approved by the Institutional Review Board. From each patient, 8 mL of peripheral blood was collected in a Rare Cell blood collection tube (Streck, Omaha, NE) and processed within 24 hours.

### 2.2. Immunofluorescent Staining Protocol for Labeling CTCs

Blood samples were subjected to an isotonic lysis with ammonium chloride buffer (155 mM NH_4_Cl, 10 mM KHCO_3_, 0.1 mM EDTA, pH 7.4). After 5 minutes on a rotator and a 5-minute centrifugation at 700 g, the supernatant containing lysed red blood cells was removed. The resulting nucleated cell pellet was resuspended in phosphate buffered saline (PBS) and distributed on custom-designed adhesive slides (Marienfeld, Germany) that have an active area of 62 cm^2^ that can hold a monolayer of roughly 27 million nucleated cells. Slides were incubated at 37°C for 40 minutes. Cells were then fixed with 2% paraformaldehyde for 20 minutes, washed with PBS, and permeabilized with cold methanol for 5 minutes, followed by another PBS wash step. Subsequently, 10% goat serum was added for 20 minutes to block nonspecific binding sites, followed by incubation at 37°C with monoclonal antipan cytokeratin (Sigma, MO) and conjugated CD45-Alexa647 (Serotec) antibodies for 40 minutes. After a PBS wash, the secondary antibody, Alexa555 (Invitrogen), was added for 20 minutes. Cells were counterstained with DAPI for 10 minutes and mounted with an aqueous mounting media.

### 2.3. FAST Scanning and ADM-Coupled Imaging of Immunofluorescently Labeled CTCs

Fiber-optic Array Scanning Technology (FAST) was used to identify the location of CTCs as described previously [25]. Briefly, an argon-ion laser excites fluorescence in labeled cells and determines the location of a fluorescently labeled cell by the scan and stage positions at the time of emission at a scan rate of 25 million cells per minute. Each FAST-identified object was then imaged with a 20X objective in fluorescence via automated digital microscopy (ADM).

### 2.4. Relocation of CTCs for Morphological Analysis

**Figure 1:**
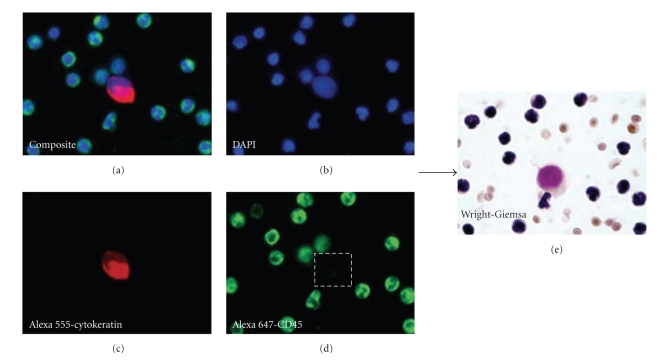
Relocation and characterization of CTCs using the Wright-Giemsa stain. After initial identification of CTCs via fluorescent images, subsequent Wright-Giemsa stain and/or Papanicolau stain of these cells is performed: (a) composite 3-color fluorescent image, (b) DAPI channel only, (c) cytokeratin channel only, (d) CD45 channel only, and (e) corresponding Wright-Giemsa-stained cell.

A pathologist used 20X magnification and applied strict criteria defining CTCs as cytokeratin positive, CD45 negative, and DAPI positive (nuclear stain). Enumeration of CTCs was done at 20X for all patient specimens. To morphologically evaluate fluorescent CTCs for further cytologic details, 100X images were taken. Subsequently, coverslips were removed and the Wright-Giemsa stain and/or Papanicolau stain was applied. Cells were then relocated using calibration fiducials and imaged in brightfield for further cytomorphologic analysis (Figure 1). 

### 2.5. Patient Analysis

Primary and metastatic tumor biopsies were reviewed by a single pathologist. Architectural and cytologic features of each primary tumor and available metastatic tumor biopsies were reviewed. Patient status at the time of the blood draw (stable disease versus progressive disease) was determined by a blinded medical oncologist review of lab data including CEA, radiology, and clinician assessment of the patient around the time of draw.

## 3. Results

**Figure 2:**
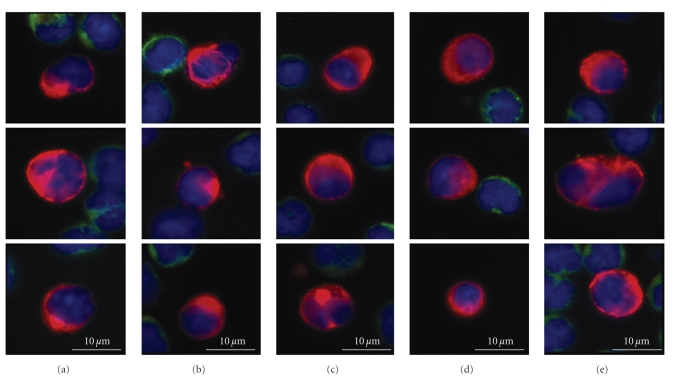
Representative CTCs found in Patients 1–5. Each column represents one patient's CTCs: Red = cytokeratin, Green = CD45, and Blue = DAPI.

Patient and tumor characteristics are summarized in Table 1. Patients varied in the number and types of metastases as well as their line of therapy; however, review of the primary tumor slides demonstrated that all had presented with moderately differentiated adenocarcinoma. When primary tissue and metastatic tissue biopsies were available, all available tissue biopsies were examined. These 5 patients had detectable CTCs ranging from 12–282 per 8 mL of blood. Although not a clinical outcomes study, we did note that patients who were identified as having progressive disease had significantly more cells than those patients who were stable at the time of the blood draw. Figure 2 shows representative CTCs found in each patient. 

### 3.1. Detailed Cytomorphologic Evaluation of CTCs

**Figure 3:**
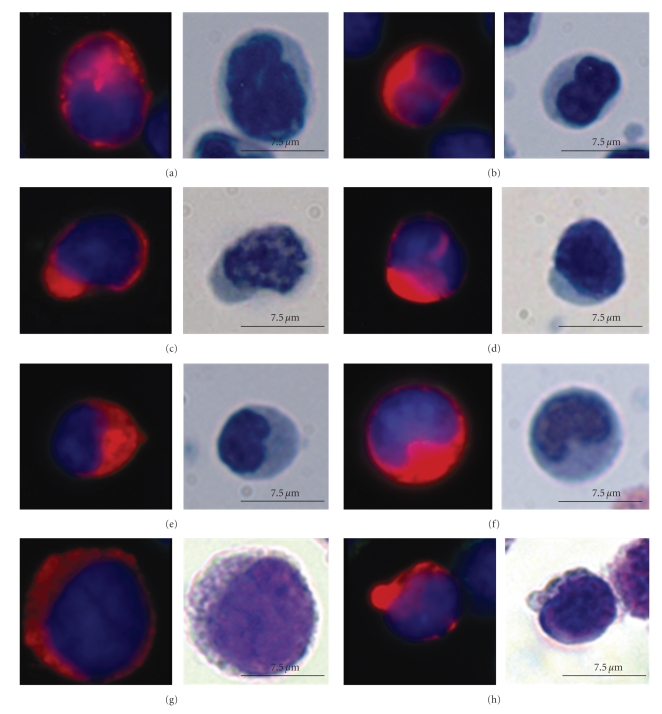
CTCs pleomorphism in the blood of metastatic colorectal patients using fluorescent, Papanicolau, and Wright-Giemsa stains. Representative types of CTCs identified in patient cohort. Left: fluorescent-stained CTC; Red: Alexa555-cytokeratin, Green: Alexa 647-CD45, and Blue: DAPI. (a)–(f) Right: corresponding CTC viewed with Papanicolau stain. (g), (h) Right: corresponding CTC viewed with Wright-Giemsa stain.

In the peripheral blood, we found a markedly pleomorphic population of colon tumor cells as shown in the gallery of colorectal CTCs (Figure 3). There were not only large, high nuclear-to-cytoplasm (N/C) ratio cells (Figures 3(a) and 3(g)) but also cells with moderate to voluminous amounts of cytoplasm, yielding moderate-to-low N/C ratios (Figures 3(e) and 3(f)). Overall CTC size also varied. Many were larger than the surrounding benign WBCs (Figures 3(a), 3(f), and 3(g)); however, there were also cells of the same size or smaller than surrounding WBCs (Figures 3(c), 3(d), and 3(e)). In some cases, the smaller cells showed features suggestive of apoptosis, such as irregular nuclear or cytoplasmic condensation or frank fragmentation into dense rounded structures (Figure 3(e)).

Notable were frequent cells with irregular nuclear contours, either large lobations or fine irregularities (Figures 3(a) and 3(b)). Some of the nuclei with large lobations appeared monocytoid in shape, or showed a half moon-like configuration (Figure 3(f)); the nuclear invagination is occupied by cytokeratin-rich cytoplasm. In other cells, the nuclear irregularities are on a smaller scale, yielding a finely irregular nuclear contour to a generally round or oval nucleus (Figure 3(c)); these features are best demonstrated by high-powered examination of Papanicolau-stained cells at the microscope.

Eccentricity of the nuclear location within the cell cytoplasm is also a prominent feature in many of the circulating colon cancer cells. While the cytoplasm extends circumferentially around the nuclei, many cells show a cytoplasmic bulge on one side, occupied by cytokeratin-rich cytoplasm (Figures 3(c) and 3(d)). The corresponding Wright-Giemsa and Papanicolau-stained cells demonstrate a “plasmacytoid” appearance familiar to cytopathologists examining cytologic preparations of various adenocarcinomas.

### 3.2. Detailed Architectural, Histologic, and Cytomorphologic Review of Primary/Metastatic Tumor

**Figure 4:**
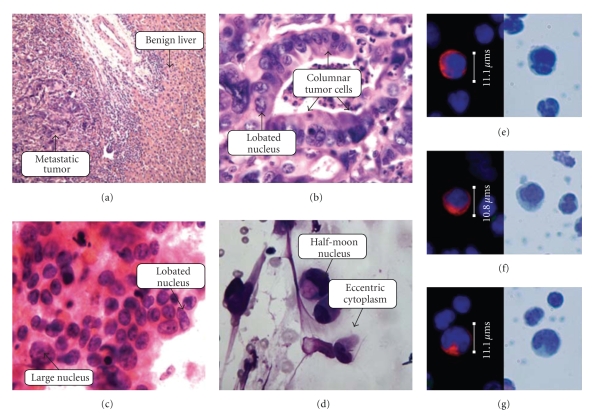
Comparison of CTC morphology with biopsy histology and touch preparation cytology in a patient with metastatic colorectal cancer. (a) Low-power histology of the liver metastasis from 1999 of Patient E (H & E, 20X). (b) High-power histology of the liver metastasis from 1999 of Patient E (H & E, 110X oil). (c) High-power cytology of retroperitoneal metastasis from 2002 of Patient E (H&E Stain, 100X oil). (d) High-power cytology of retroperitoneal metastasis from 2002 of Patient E (Wright-Giemsa stain, 100X oil). (e)–(g) CTCs identified in the blood of Patient E in 2008.

All primary tumors in this small series were classified as moderately differentiated, with architectural features showing a predominance of abnormal gland formation and only focal areas of sheet-like growth. Figure 4 shows histology, cytology, and representative circulating tumor cells found in Patient E. This histology is representative of all patients in our series. The glandular lumens often contain necroinflammatory debris, the familiar “dirty necrosis” of colon cancer. The abnormal glands are formed by columnar cells with nuclear enlargement, hyperchromatism, elongation, and stratification. In some of the less well-differentiated glands, the tumor cells approach a cuboidal shape, but overall the predominant tumor cell shape is columnar. Individual cells vary in size; although most cells of the tumor are larger than any surrounding benign cells, there is a marked range of cell size within the tumor, and cells at the smaller range of cell size approach that of neighboring histiocytes or other benign stromal cells. N/C ratio is also quite variable within the tumor cell population; while high N/C ratio cells are certainly present, there are also abundant cells with moderate-to-low N/C ratios due to their voluminous cytoplasmic domain. Individual tumor nuclei also frequently show nuclear lobation, and/or fine nuclear irregularities, features more easily appreciated in the cytology specimens from fine needle biopsies of metastases (Figure 4). Apoptotic or degenerating individual tumor cells are frequently observed, as are mitotic figures.

### 3.3. Cytomorphologic Comparison of CTCs to the Primary and Metastatic Tumors

In comparison to the tissue biopsies from both primary and metastatic tumor sites, the morphologic cell mix of circulating colon tumor cells is similar in its pleomorphism. Certain cytologic features noted in the tissue biopsies, in particular prominent lobation of nuclear contours, are clearly evident in the circulating population. As well, fine irregularity of nuclear membrane contours noted in solid tissue samples persists in the circulating population of tumor cells. Finally, colon tumor cells in circulation show a morphologic tendency toward eccentricity of the cytokeratin-rich cytoplasm, while their solid tissue counterparts appear columnar in shape.

## 4. Discussion

Comparison of primary and metastatic colon tumor cells to circulating colorectal tumor cells shows marked congruence of cytologic features between the tissue compartments as illustrated in Figures 2–4. Histopathologically, these tumors are moderately differentiated adenocarcinomas, with columnar tumor cells forming abnormal glands; the nuclei are irregularly contoured and frequently lobated. Cytologically, the primary and metastatic tumor deposits show pleomorphism among the individual cells of the tumor tissue. Circulating cells from these same patients retain this cytomorphology, showing overall pleomorphism with variations in size, shape, and N/C ratios as well. The CTCs show nuclear contour irregularity and lobation, with eccentrically located cytoplasm corresponding to a columnar-type adenocarcinoma cell. This analysis supports the premises that CTCs retain primary tumor cytologic characteristics and that CTCs represent a random sampling of the many phenotypic cell types present in the primary and metastatic tumor deposits. The findings argue against the theory that only particular subsets of tumor cells enter the peripheral blood, such as very poorly differentiated “stem cell”-like tumor cells, or only visibly dead/dying apoptotic tumor cells. The findings support the possibility that circulating tumor cells consist of a combination of passively “shed” cells with little malignant potential and actively “migrating” cells that are viable and may go on to form further metastatic foci [26].

In comparison to a previous morphologic study of CTCs in breast cancer, the pleomorphism in the circulating population of colorectal cancer is similar to the pleomorphism in the circulating population in breast cancer. However, there appear to be subtle, but appreciable cytologic differences between the two cancer types; the circulating breast cancer cells are generally round with circumferential cytoplasm, while many of the circulating colon cancer cells appear eccentric, with a cytoplasmic bulge on one side. The subtle differences in shape between CTCs of the two tumor types are felt likely to be a result of the cell shape of the original tumor type. The cells of breast cancer are usually cuboidal rather than columnar, and the corresponding detached CTCs show evenly distributed cytoplasm with no discernible eccentricity. One can theorize that the more exaggerated the columnar shape and/or basally oriented nuclei of the original tumor cells, the more eccentric the cytoplasm will appear when the cell detaches from the stroma and enters the circulation. Future evaluation of additional cases of adenocarcinoma where the primary tumor is either very well differentiated or very poorly differentiated will serve to further address this connection between the appearance of the cells in tissue versus that in circulation.

Morphologic evaluation of the characteristics of circulating tumor cells from different malignancies including patients with nonmetastatic disease will contribute to our future understanding of the metastatic process. Furthermore, as CTC enumeration and characterization enter the mainstream of cancer diagnostics and care, familiarity with the appearance and variability of features of CTCs will be important for pathologists. This small series demonstrates that colon cancer cells in circulation are not dissimilar in appearance from the cells in tissue within a single patient, and that between tumor types, the cells in circulation may vary in their appearance. As new techniques for further assessment of the circulating component of epithelial tumors emerge, these cells may serve as an easily accessible real-time ‘biopsy’ of the active tumor in a patient's body.

## Figures and Tables

**Table 1:** Patient tumor characteristics and circulating tumor cell count.

Patient	Age/sex	Time since diagnosis (mon)	Primary tumor site	Locationof metastases	Line of therapy	CTCs (no. per 8 mL)	Patient status
Patient 1	60/M	75	Colon	Liver, LN, lungs, bone, paraspinal	7th	282	Progressive

Patient 2	54/M	37	Colon	Liver	4th	25	Stable

Patient 3	70/F	27	Colon	Liver	1st	12	Stable

Patient 4	44/F	16	Rectum	Liver, lungs, LN ovaries, retroperitoneum	1st	23	Stable

Patient 5	66/F	87	Colon	Liver, retroperitoneal, lung, endometrium	9th	164	Progressive
